# The difficult coughing child: prolonged acute cough in children

**DOI:** 10.1186/1745-9974-9-11

**Published:** 2013-04-10

**Authors:** Michael D Shields, Surendran Thavagnanam

**Affiliations:** 1 Respiratory Medicine, Royal Belfast Hospital for Sick Children, Centre for Infection & Immunity, Queen’s University Belfast, Health Sciences Building, 97 Lisburn Road, Belfast, Bt7 9BL, N Ireland, UK; 2 Department of Paediatric Respiratory Medicine, Faculty of Medicine, University of Malaya, Kuala Lumpur, Malaysia

**Keywords:** Prolonged acute cough, Children

## Abstract

Cough is one of the most common symptoms that patients bring to the attention of primary care clinicians. Cough can be designated as acute (<3 weeks in duration), prolonged acute cough (3 to 8 weeks in duration) or chronic (> 8 weeks in duration). The use of the term ‘prolonged acute cough’ in a cough guideline allows a period of natural resolution to occur before further investigations are warranted. The common causes are in children with post viral or pertussis like illnesses causing the cough. Persistent bacterial bronchitis typically occurs when an initial dry acute cough due to a viral infection becomes a prolonged wet cough remaining long after the febrile illness has resolved. This cough responds to a completed course of appropriate antibiotics.

## Prolonged acute coughing in children

Cough in children is the most common presenting symptom to general practitioners and persistent cough is commonly referred to paediatricians for further investigation and treatment [1]. The cough can be very distressing for parents to watch especially if it interferes with daily activities and often disturbs both the parents and child’s sleep [2]. While coughing may be seen as a mere troublesome symptoms without any serious consequences, ignoring cough that may be the sole presenting symptom of an underlying respiratory disease may lead to delayed diagnosis and progressions of a serious illness or chronic respiratory morbidity. In most children acute coughing is usually due to a viral upper respiratory tract infection (URTI) such as a simple head cold with bronchitis or croup. Less often, but still common, pathogens can involve the lower respiratory tract system causing bronchiolitis, whooping cough, or pneumonia. Symptomatic URTI with cough in school children typically occurs around 7–10 times per year.

### When does an acute cough typically end

The majority of children with acute coughing with a simple head cold have an associated bronchitis and the coughing typically abates by 10 to 14 days. In one study parents assessed the cough as moderate or severe in more than 80% cases and the coughing to be frequent or continuous in more than 70% cases [3]. The longest cough duration in this study was 21 days and initially more than 50% of parents described the cough as dry with the remainder reporting the cough to be productive or of a mixed type. Recently Mitra *et al.* followed the course of acute URTI in children and reported cough to be the second commonest symptom to runny nose occurring in more than 80% of children [4]. The coughing occurred after an initial 1–2 days of systemic illness with fever and a feeling of un-wellness. The coughing lasted a median of 5 days and in this study, all children had stopped coughing by 20 days. Prospective studies of acute cough in young children in general practice have suggested that about 50% recover by 10 days and 90% by 3 weeks, so 10% of children still have problems in the third to fourth weeks [5,6]. This is supported by a recent systematic review of the natural history of acute cough in which it was estimated that about one-quarter will still be unwell with cough at 2 weeks [7].

### When should a cough be called ‘chronic’

No studies have clearly defined when a cough should be labelled as chronic. It has been recognised in adult studies that many patients whose chief complaint was cough lasting for more than 3 weeks the cough usually resolved spontaneously without any treatment. However, spontaneously resolving cough was exceedingly rare in adults who had experienced a long duration of cough such as for several months or years [8]. Thus patients with cough of a relatively short duration must be regarded separately from patients with a cough of a longer duration.

The BTS Recommendations for the assessment and management of cough in children defined chronic cough as a cough which had lasted longer than 8 weeks rather than the 4 weeks recommended in the American College Chest Physicians (ACCP) guidelines [9,10]. The thinking behind the decision to include an intermediate time zone defined as ‘prolonged acute cough’ in this guideline was to allow a period for cough resolution for the 10% of normal children who are still coughing with a simple head cold after 2–3 weeks. If the child is otherwise normal and the cough is resolving no further investigations would be indicated. Warnings were included that a ‘wait and see’ policy was not recommended if a retained inhaled foreign body is considered a possibility, if the child has already signs of chronic lung disease or when the coughing is progressively becoming worse (e.g. consider pertussis, retained inhaled foreign body, expanding mediastinal neoplasm, lobar collapse secondary to mucus plug and tuberculosis (often with accompanying weight loss). Most children with a prolonged acute cough were thought to have a post viral syndrome or a pertussis like illness. This approach mirrors the adult recommendations where a cough lasting more than 8 weeks was defined as chronic and a cough lasting more than 3 weeks but resolving by 8 weeks was called a subacute cough [11]. On the other hand, the Australian and American College Chest Physicians (CACP) guidelines on cough in children defined chronic cough as a cough that lasts longer than 4 weeks [10,12]. Here the authors observed only 13.9% of the total 346 children’s’ cough had resolved without any specific diagnosis and the remaining primary aetiology observed needed medical investigations. The authors also observed no differences in duration of cough or cough score in children with serious underlying disease compared to those with less serious conditions.

This approach theoretically might encourage earlier and possibly unnecessary investigations. However, it may highlight to primary care physicians and paediatricians the need to consider earlier the treatment of persistent bacterial bronchitis (see below). A recent RCT has confirmed the benefits of a 2 week course of amoxicillin-clavulanate in children with a prolonged wet or productive cough lasting more than 3 weeks [13].

### What are the causes of prolonged acute cough in children

There is limited information regarding the causes and clinical courses of prolonged acute cough in the paediatric literature. It is important to remember that the causes of chronic cough in children must have started at some time and gone through the prolonged acute coughing phase and need to be thought of but chronic cough is not the focus of this article. In addition, many children experience recurrent acute cough/prolonged acute cough which parents will not readily distinguish from chronic cough. The causes of and approach to chronic coughing has been clearly described in guidelines (9,10). Therefore, our intention is to highlight some of the possible causes and clinical courses of prolonged acute cough where complete resolution is to be expected.

#### Aetiology of prolonged acute cough

The most common cause for prolonged acute cough in children is post viral or post-infectious cough. Post-infectious cough can be defined as a cough that began with symptoms related to the common cold and persists. It has a high rate of spontaneous resolution without any therapeutic intervention.

Some specific causes of prolonged acute coughing are as follows:

#### Infants with acute bronchiolitis

Acute bronchiolitis is a common acute respiratory infection especially in children less than 1 year. The children clinically present with tachypnoea, crackles, dry cough and audible wheeze. The symptoms typically worsen in the acute phase of bronchiolitis before resolving by 14 days. Although bronchiolitis is usually a self-limiting condition, a significant number of children have persistent respiratory symptoms such as cough in the post-acute phase [14].

Indeed, a generally dry, irritating cough is the most common symptom in bronchiolitis (98% of Respiratory Syncytial Virus (RSV) positive infants and causes significant levels of concern to parents of children affected [14,15]. Cough is recognised by parents with a less variable interpretation than other potential markers (increased work of breathing, wheezing) and so reduction in cough duration would be considered an important benefit.

Systematic reviews looking at the therapeutics strategies in reducing the morbidities following acute bronchiolitis showed that neither the use of inhaled glucocorticoids or leukotriene antagonists during acute bronchiolitis prevent post-bronchiolitic wheezing or cough [16].

#### Pertussis infection

While infants too young to have been vaccinated are at particular risk for severe whooping cough disease there has been a recent epidemic of pertussis as a cause of prolonged acute coughing in older children and adolescents in many countries [17].

**Figure 1:**
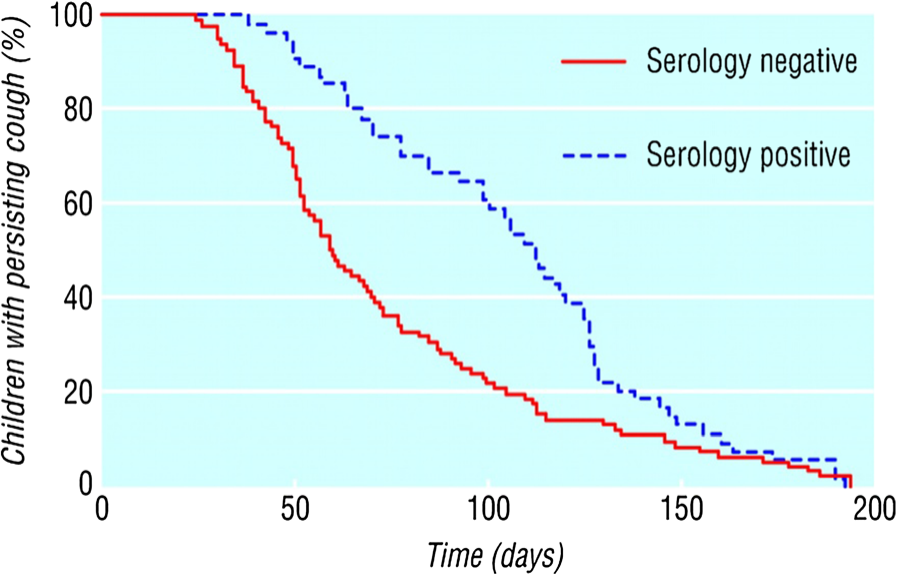
**Proportion of children continuing to cough each day after onset according to serology.** Reproduced with permission [20].

In a non-outbreak setting, Cornia et al. determined that 32% of prolonged acute cough was due to pertussis and that the diagnosis needs to be considered even when the classical pertussis symptoms are not present [18].They performed a systematic evaluation of the utility of traditional signs of pertussis. Paroxysmal cough had a sensitivity of 86% and specificity of 26%, post-tussive whoop had a sensitivity of 50% and specificity of 73% and post-tussive vomit had a sensitivity of 70% and specificity of 61%. The presence or absence whooping or vomiting only modestly increased the likelihood of pertussis. Because the current peak in one study was in children aged 8–11 years, the authors speculated that older children remained better protected as they had received whole cell vaccine which was in widespread use for infants until the late 1990’s [19]. The newer acellular vaccines may not protect children for as long as the older whole cell vaccine and the newer vaccine was introduced at the same time as media scares may have reduced uptake. In a community study that recruited children (5–16 years of age) coughing for longer than 2 weeks, 37% of them had serological evidence of a recent pertussis infection and the median duration of coughing was 112 days (range: 38 to 191 days) [20,21]. Those children who were negative to pertussis (many with mycoplasma infection) also had prolonged coughing but this was shorter than the pertussis group (median duration 58 days, range 24 to 192 days). Virtually all children in this study had complete resolution of the cough (Figure 1). It is thus important to note that if a trial of treatment such as inhaled corticosteroids (ICS) had been started the ICS would have appeared to have worked but the resolution would have been due to the natural resolution that occurs.

Treatment with a macrolide antibiotic may be beneficial in pertussis but only when administered in the early stages of the disease. This is difficult to implement because the diagnosis is often not thought about until the cough has become chronic unless there is known contact with an index case. It is recommended to only treat patients aged above 1 year within 3 weeks of cough onset and infants aged less than 1 year within 6 weeks of cough onset [22]. Antibiotics decrease the duration of infectiousness and thus prevent spread [23]. If the patient is diagnosed late, antibiotics will not alter the course of the illness and, even without antibiotics, the patient should no longer be spreading pertussis.

#### Children recovering from a complicated acute pneumonia (e.g. empyema)

At least a third of children who initially have a treated empyema are still coughing by 4 weeks with one quarter at 6 months reducing to around 3% at 12 months. Some of these patients have prolonged cough due to residual of disease and as a result will benefit from a prolonged course of antibiotics 1–4 weeks from discharge or longer [24,25].

#### Rhinosinusitis

The criteria used to diagnose rhinosinusitis in children are nasal secretions with or without a wet or dry cough occurring longer than 10 days. Chronic rhinosinusitis is more common in those with atopy and is considered present if symptoms persist longer than 4–8 weeks. Facial pain and discomfort is not so common in children when compared with adults.

Antibiotics are generally recommended for acute bacterial sinusitis but two of the four placebo controlled clinical trials were negative. This may have resulted from including those with allergic rather than an infective cause or an inappropriate antibiotic dosage. In the other two studies using amoxicillin-clavulanate showed considerable benefit although at the cost of increased side-effects [26,27].

#### Retained inhaled foreign body

Foreign body aspiration (FBA) is most commonly seen in children below 24 months [28]. The diagnosis should be suspected if there is a history of choking followed by prolonged cough and non-resolving pneumonia. The yield from physical examination and radiological studies in the diagnosis of FBA is relatively low but is increased when the presentation is delayed and when history is doubtful. The sensitivity and specificity for each diagnostic criterion are as follows: clinical history (63% and 32%), symptoms (68% and 53%), physical examination findings (70.5% and 63%), radiological findings (73% and 68%) and the triad of cough, wheeze and diminished breath sound (88% and 51%) respectively [29]. Delayed diagnosis may be related to an unobserved aspiration event or lack of physician awareness and has serious consequences such as chronic cough, recurrent pneumonias and eventually localised areas of bronchiectasis. The immediate management is endoscopic removal of the foreign body and this should be done in case where there is parental or clinical suspicion.

#### Persistent bacteria bronchitis

Persistent bacterial bronchitis (PBB) has been defined as the presence of a chronic wet cough with resolution of cough with appropriate antibiotics and absence of pointers suggestive of alternative specific cough [30,31] Recently, an association between PBB starting in infancy and airway malacia (tracheal, bronchial) has been described [32]. The cough of PBB resolves after a course of antibiotic such as amoxicillin-clavulanate for 2 weeks but some require a longer 4–6 weeks antibiotic. If PBB fails to respond to antibiotics or if PBB becomes recurrent, then further investigations are required to rule out the other conditions such as subtle immunodeficiencies or other causes of chronic suppurative lung disease [33]. The long term natural history of PBB is unknown. It has been speculated that it might be a precursor for chronic suppurative lung disease with formation of bronchiectasis but could also be a fore runner for adult chronic obstructive pulmonary disease. However, if children with immune deficiencies are excluded, PBB is associated with an augmented rather than deficient innate immune system [34]. It is important not to forget that persistent endobronchial infections occur in other conditions known to cause chronic coughing including cystic fibrosis, immune deficiencies, primary ciliary dyskinesia and recurrent pulmonary aspiration.

## Conclusions

A significant minority of children cough for longer than 3 weeks after a simple viral head cold. If the child is otherwise well and the cough is dry and there are no specific alerts for a serious disease and the cough is resolving a period of observation is all that is recommended. Investigations are required earlier if there is a suspicion of a retained inhaled foreign body, the cough is progressively worsening or there are already signs of chronic disease present. If a child develops a prolonged wet cough after a head cold has resolved antibiotics should be considered as the child could have persistent bacterial bronchitis or rhinosinusitis. Pertussis has increasingly been identified as a cause of prolonged acute cough in older children who may have an atypical cough and the children may have been previously vaccinated with the acellular vaccine. The natural history of the cough with pertussis or a post viral syndrome is natural resolution. When trials of anti-asthma therapy is used care must be taken not to mistake natural resolution as response to the therapy.

## Competing interests

Neither MDS nor ST makes any declaration of interest for this review. MDS chaired the production of The BTS Recommendations for the assessment and management of cough in children guidelines.

## Authors’ contribution

MDS and ST co-wrote this review article using the literature and their personal clinical experience. All authors read and approved the final manuscript.
